# The Relationship Between Big Five Personality Traits and Driving Styles in Older Adults

**DOI:** 10.1093/geroni/igaa057.1256

**Published:** 2020-12-16

**Authors:** Junyan Tian, Anabella Raika, Despina Stavrinos, Lesley Ross

**Affiliations:** 1 Penn State University, State College, Pennsylvania, United States; 2 University of Alabama Birmingham, Birmingham, Alabama, United States

## Abstract

Older adults’ psychosocial factors, including personality, are correlated with driving performance and driving cessation. However, the relationship between personality and driving styles has been examined only among young and middle-aged drivers. This study examined the relationships of personality factors and self-reported driving styles among 72 healthy older drivers aged 65-85 (M=72.29, SD=5.36) using the Multidimensional Driving Style Inventory (MDSI) scale to measure reckless and careless, anxious, angry and hostile, and patient and careful driving styles. Personality was accessed with the Big Five Personality questionnaire. Correlational results indicated that less conscientiousness was significantly correlated with increased reckless and careless and less patient and careful driving styles; and lower agreeableness was significantly correlated with greater angry and hostile and less patient and careful driving styles. Being a man was associated with greater reckless and careless and angry and hostile driving styles. Age was not associated with driving styles. Accordingly, three regressions were tested. After controlling for gender, only lower conscientiousness was associated with greater reckless and careless driving style (β=-.007, p=.03). Men had a higher risk of reckless and careless (β=.342, p<.01) and angry and hostile (β=.392, p<.01) driving styles. Our results highlight the relationship between personality traits and self-reported driving styles among older adults, and how gender may influence some of these relationships. Future research should further investigate the associations between gender and personality traits and older adults’ driving mobility and safety.

